# Impact of Routinely Performed Optical Coherence Tomography Examinations on Quality of Life in Patients with Retinal Diseases—Results from the ALBATROS Data Collection

**DOI:** 10.3390/jcm12123881

**Published:** 2023-06-07

**Authors:** Alexander K. Schuster, Christian Wolfram, Tobias Hudde, Alexander Klatt, Birthe Schnegelsberg, Heven Midani-Oezkan, Mike Ross, Focke Ziemssen, Norbert Pfeiffer

**Affiliations:** 1 Department of Ophthalmology, University Medical Center, Johannes Gutenberg-University Mainz, Langenbeckstr. 1, 55131 Mainz, Germany; 2 Department of Ophthalmology, University Medical Center Hamburg-Eppendorf, Martinistr. 52, 20246 Hamburg, Germany; 3 Eye Hospital Wolfsburg-Fallersleben, Am Spieker 10, 38440 Wolfsburg, Germany; 4 Eye Center Klatt, Henry-Wetjen-Platz 3, 28844 Weyhe, Germany; 5 Novartis Pharma GmbH, Roonstr. 25, 90429 Nuremberg, Germany; 6 Department of Ophthalmology, Leipzig University Hospital, University of Leipzig, Liebigstraße 12, Haus 1, 04109 Leipzig, Germany; 7 Center for Ophthalmology, Eberhard-Karls-Universität Tübingen, Elfriede-Aulhorn-Str. 7, 72076 Tübingen, Germany

**Keywords:** optical coherence tomography, age-related macular degeneration, diabetic macular edema, branch vein occlusion, central retinal vein occlusion, health-related quality of life, visual acuity, ranibizumab, aflibercept

## Abstract

The use of OCT to monitor intravitreal treatment varies in clinical practice and is not always mandatory. The ALBATROS data collection aimed to clarify the impact of routinely implemented OCT on clinical outcomes and its impact on vision-related quality of life (VRQoL). Methods: An observational cohort study included patients with retinal diseases starting an intravitreal anti-vascular endothelial growth factor treatment in Germany. Treatment followed clinical practice except mandatory OCT examination during the 12-month observation period. VRQoL was assessed by NEI VFQ-25 and compared with respect to OCT examinations and number of intravitreal injections in the different diseases (nAMD, DME, BRVO, CRVO). Results: 1478 patients (74.5 ± 10.9 years, 54.9% female) were included in the analysis. Patients had neovascular AMD (65.2%), DME (18.4%), BRVO (9.5%), or CRVO (6.9%). 8.8 ± 2.6 OCT examinations and 6.1 ± 3.2 intravitreal injections were performed within 12 months. VRQoL differed between indications at baseline, with substantially lower values for neovascular AMD and CRVO. After twelve months, an increase in visual acuity and visual functional scale was observed for nAMD, DME, and BRVO, while in DME only, there was an association between number of OCT examinations and VRQoL. Conclusion: Intravitreal treatment was able to maintain VRQoL over twelve months in a real-world setting. Regular OCT examinations were associated with higher gain in VRQoL in DME patients after 12 months.

## 1. Introduction

Randomized clinical trials have promoted the safety and efficacy of anti-vascular endothelial growth factor (VEGF) treatments in patients with retinal diseases [1,2,3]. The anti-VEGF treatments ranibizumab (Lucentis^®^) and aflibercept (Eyelea^®^) were approved in Germany for the treatment of retinal diseases, including neovascular age-related macular degeneration (nAMD), diabetic macular edema (DME), branch retinal vein occlusions (BRVO), and central retinal vein occlusions (CRVO). To increase treatment success, national treatment guidelines along with recommendations for disease monitoring were regularly updated to include different treatment regimes. After an initial 3-month upload phase with monthly anti-VEGF injections, treatment regimens now comprise both fixed schemes, including regular eye examinations and injections, as well as variable schemes. In this context, differences in maintaining visual improvement could be observed between pivotal RCTs and clinical practice [4], with less improvement in visual acuity in clinical practice, which is suggested to be due to lesser stringent monitoring and care of patients [5].

Before 2019, national treatment guidelines recommended regular OCT examinations at monthly intervals but were hindered by the lack of uniform reimbursement by German statutory health insurance (SHI) providers, and consequently, OCTs were not routinely performed in clinical practice.

Thus, the actual impact of OCT examinations on treatment success remains elusive. The OCTAVE study, which investigated the impact of OCT on treatment success, was prematurely terminated. The statutory health insurance providers decided in 2019 to include OCT in their benefits catalog [6].

Besides clinical outcomes, vision-related quality of life (VRQoL) has been frequently used to evaluate treatment success. To this end, the 25-item National Eye Institute Visual Functioning Questionnaire (NEI VFQ-25) served previously as a validated questionnaire assessing VRQoL in patients with visual impairment. However, as composite scores are reported to be impacted by multidimensionality and subscales to be psychometrically unsound, NEI VFQ-25 was therefore revised in terms of redefining new Rasch-transformed dimension scores, i.e., visual functional scale (VSF) and socioemotional scale (SES) [7].

The present study aims to investigate the impact of mandatory OCT examinations on treatment outcomes and especially VRQoL in patients with retinal diseases receiving an approved anti-VEGF treatment in clinical practice.

## 2. Materials and Methods

ALBATROS study design: The ALBATROS study has been designed to evaluate the impact of mandatory OCT examination in clinical routine work in treatment-naïve patients receiving intravitreal injection therapy for retinal diseases. Hence, data collection was performed in Germany between January 2016 and September 2019. Intravitreal anti-VEGF-naïve patients diagnosed with neovascular age-related macular edema, diabetic macular edema, branch retinal vein occlusions, or central retinal vein occlusions for which any approved anti-VEGF treatment was indicated, were included. Treatment and diagnostics were conducted according to the national guidelines of expert associations for the particular indications, except for performing a mandatory OCT examination at each control visit, which was hitherto not routinely performed due to missing reimbursement by health insurance. 

The collected data included information about best-corrected visual acuity (BCVA), central retinal thickness (CRT) as assessed by OCT, number of anti-VEGF injections and performed OCT scans, quality of life assessing health (SF-9), and VRQoL using the 25-item National Eye Institute Visual Functioning Questionnaire (NEI VFQ-25). Clinical data were collected prospectively at baseline and retrospectively at a second visit twelve months later, covering patients’ clinical data of the past year. Quality of life was assessed at baseline and 12-month visits. 

ALBATROS data were analyzed in total and stratified by indication (nAMD, DME, BRVO, and CRVO). No adjustments for multiplicity were applied and the data should be considered descriptive. Best-corrected visual acuity (BCVA) at baseline was assessed separately for the study eye and the fellow eye by means of EDTRS letters. The study eye was defined as the one for which intravitreal operative medication injection (IVOM) with an anti-VEGF treatment was initiated. The better eye was defined as the one that shows higher visual acuity at baseline compared to the other. Patient demographics and QoL assessments were analyzed for the better eye, i.e., including eyes not necessarily being subject to anti-VEGF therapy; depending on the particular indication, the fellow eye was the better eye in about 60–90% of the patients (nAMD: 66.7%; DME: 63.2%; BRVO: 87.9%; CRVO: 88.2%). Changes in BCVA and central retinal thickness (CRT) after twelve months from baseline were presented for the study eye. A linear regression model was used to estimate the dependency of changes in BCVA at the last visit or in QoL and OCT examination frequency; p-values denote the association between the predictor variable and changes in the response variable. Vision-related QoL assessed by NEI VFQ-25 was analyzed according to Pesudovs et al. [7], showing Rasch-transformed long-form visual function scale (LFVFS25; including questions 2, 5 to 10, and 14) and long-form socioemotional scale (LFSES25; including questions 11, 13, 17, 18, and 20 to 25). The ALBATROS study was registered at the German Clinical Trials Register (DRKS) under DRKS-code: DRKS00010267.

OCEAN study design: The prospective, non-interventional OCEAN (Observation of treatment patterns with LuCEntis and real-life ophthalmic monitoring, including optional OCT in Approved iNdications; NCT02194803) study was performed in Germany between December 2011 and December 2016. Study design, patient demographics, and baseline NEI VFQ-25 have been published previously [5,8]. Briefly, patients diagnosed with nAMD, DME, BRVO, CRVO, or choroidal neovascularization secondary to pathologic myopia, being naïve for intravitreal steroid treatment, not receiving any anti-VEGF treatment within the last three months before enrollment, and for whom therapy with the anti-VEGF treatment ranibizumab was indicated, were included into the study. Besides the evaluation of changes in visual acuity after twelve and 24 months (primary endpoints), vision-related quality of life was assessed by the NEI VFQ-25 [8]. 

Analyses of ALBATROS data and comparative analyses with OCEAN data: For analyses of ALBATROS data, a modified Full-Analysis Set, which included only patients of the FAS with a regular final 12-month visit was used. 

For comparative analyses of the baseline and 12-month data from ALBATROS and OCEAN, propensity score-matched analysis sets were created, taking age, sex, primary indication, baseline BCVA of the study eye, hypertension, and pre-treatment status (i.e., treatment naïve) into account. Overall, 1478 observations from ALBATROS and 3084 from OCEAN were read, yielding 956 1:1 pairs with best congruency. Results from the logistic regression model for propensity score matching are provided in Table S1.

## 3. Results

The ALBATROS data collection was performed at 108 sites across Germany and enrolled 3224 patients, of which 2266 were included in the Full-Analysis Set (FAS) and 1478 in the Completed Analysis Set (CAS). Most of the patients included in CAS were diagnosed with nAMD (n = 964; 65.2%), followed by patients with DME (n = 272; 18.4%), BRVO (n = 140; 9.5%), and CRVO (n = 102; 6.9%). Further details are provided in the STROBE flow chart in Supplementary Figure S1.

Mean age of the entire study population was 74.5 ± 10.9 years, and the proportion of female patients was 54.9%. The best-corrected visual acuity (BCVA) of the study eye was 56.1 ± 19.6 EDTRS letters and central retinal thickness (CRT) was 388.7 ± 150.3 µm. Demographic parameters and baseline characteristics varied among the different indications, with patients diagnosed with nAMD being older, more likely to be female, and having worse BCVA. On the other hand, patients diagnosed with DME were younger, more likely to be male, and had better BCVA (Table 1).

On average, patients received approximately six injections and underwent nine OCT examinations over the 12-month observation period, with minimal variation across indications. At least 75% of patients received a minimum of seven OCT examinations for each indication during the observation period.

**Figure 1:**
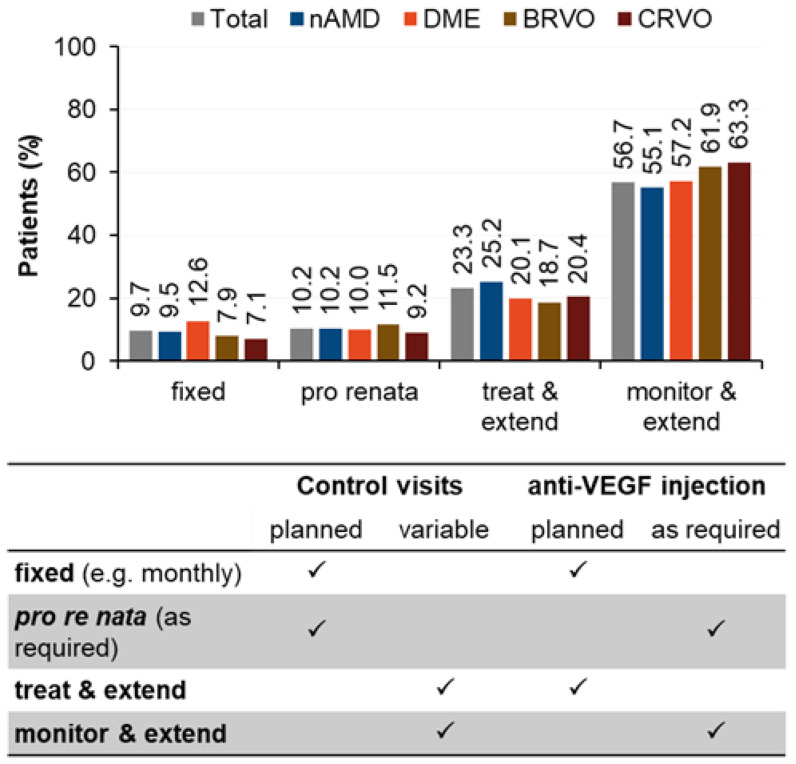
Actual performed treatment scheme at final visit.

After twelve months, most patients (about 80%) ended with “treat and extend” (TE) and “monitor and extend” (ME) treatment regimens, implying variable control visit schedules, and for the ME regimen, also variable IVOM schedules (Figure 1). On average and across all indications, 57.1 ± 30.6 days elapsed between two control visits. 

**Figure 2:**
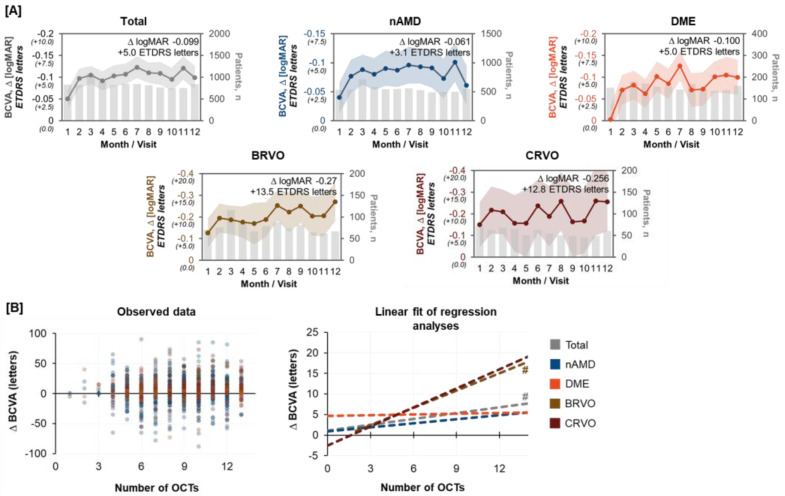
Best-corrected visual acuity of the study eye * (**A**) and association of OCT frequency and BCVA (**B**). * Study eye defined as the eye receiving IVOM with anti-VEGF treatment; it was not necessarily the worse eye at baseline. Grey vertical bars in (**A**) show number of patients for which data was available at the specified visit window, lines with dots indicate BCVA, and colored shadings show 95% confidence interval bands of respective mean BCVA values. Hashes (#) in (**B**) indicate statistical significance. | ^#^ BRVO, *p* = 0.025 (slope [95% CI]: 1.41 [0.18; 2.64]); Total, *p* = 0.015 (slope [95% CI]: 0.47 [0.09; 0.85]) with p-values denoting the association between the predictor variable (OCTs) and changes in the response variable (BCVA).

For all indications, the BCVA of the study eye showed improvement over time, with highest gains observed after twelve months of anti-VEGF therapy for patients with retinal vein occlusions (nAMD, +3.1 letters; DME, +5.0 letters; BRVO, +13.5 letters; CRVO, +12.8 letters, Figure 2A). Regression analyses were conducted to examine the impact of OCT frequency on changes in BCVA. The results showed no association for patients with DME, a low association for patients with nAMD, and a high association for patients with BRVO and CRVO (Figure 2B). However, a statistically significant association (*p* < 0.05) between the number of OCT examinations and change in BCVA was only observed for patients with BRVO and the overall study population.

**Figure 3:**
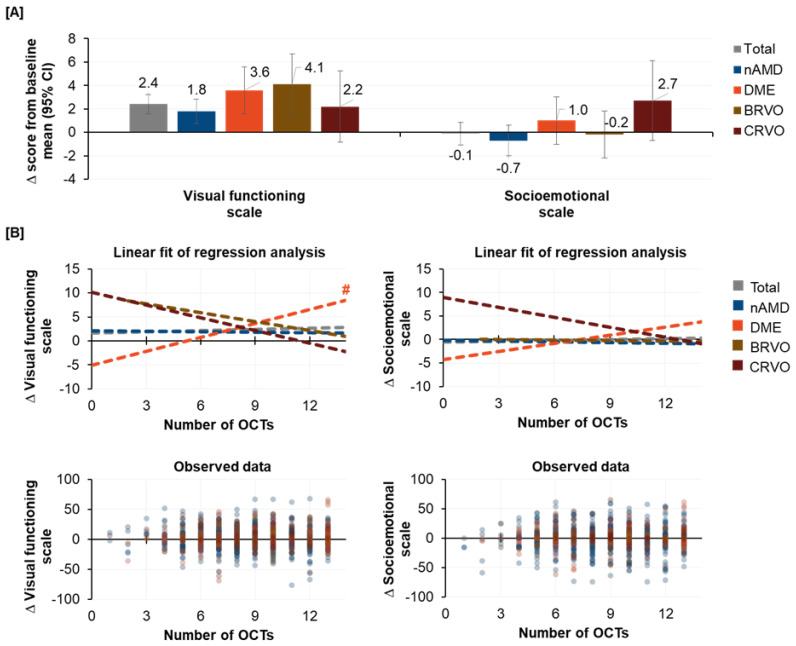
Changes in NEI VFQ-25 VFS and SES after twelve months (**A**) and association of OCT frequency on change in NEI VFQ-25 VFS and SES (**B**). Higher scores indicated higher QoL. Hashes (#) indicate statistical significance. | ^#^ VFS (DME): *p* = 0.007, (slope [95% CI]: 0.96 [0.27; 1.66]) with p-values denoting the association between the predictor variable (OCTs) and changes in the response variable (VFS).

The NEI VFQ-25 visual functional scale (VFS) and socioemotional scale (SES) were used to assess the vision-related quality of life (VRQoL). At baseline, both VFS and SES varied between the indications, with patients with nAMD and CRVO showing substantially lower values than patients with DME or BRVO (Table 1). After twelve months, slightly higher numerical values were observed for VFS across all indications, with the highest gains observed for DME (3.6 points corresponding to 5.1% gain [related to baseline]) and BRVO (4.1 points corresponding to 5.5% gain [related to baseline]). Almost no changes were observed for SES for all indications, except CRVO, which showed slight improvements compared to baseline of 2.7 points (corresponding to 3.4% gain [related to baseline]; Figure 3A). It is noteworthy that indication populations with a higher proportion of female patients, such as nAMD (61.1% females) and BRVO (52.1% females), had worse outcomes in SES than indication populations with a lower proportion of females, such as DME (38.6% females) and CRVO (43.1% females). 

To facilitate comparison with the historical data, additional analyses were conducted for the subscales and composite score of NEI VFQ-25, despite its reported limitations. Changes in composite score ranged from 0.6 points for nAMD to 2.3 points for DME. Notably, substantial improvements of at least five points from baseline to the final visit were observed for all indications in NEI VFQ-25 subscale ‘General vision’ (see Supplementary Figure S2).

To assess the association between routinely performed OCT examinations and VRQoL, changes in VFS and SES were compared with the number of performed OCT examinations (Figure 3B). Regression analyses on changes in VFS and SES revealed no association with OCT examination in patients with nAMD, but there was a numerical association for patients with DME, BRVO, and CRVO. Patients with DME had higher scores in VFS and SES with an increasing number of OCT examinations, while patients with BRVO and CRVO had lower scores in VFS with a higher number of OCT examinations. Patients with CRVO also had lower scores in SES with higher OCT frequency. However, statistically significant results were only observed for the association between OCT frequency and change in VFS in DME patients (*p* < 0.01). Of note, OCT dependency of VFS and SES for a particular indication cohort appears to be inversely correlated with the proportion of patients whose better eye was the fellow eye at baseline (see Table 1). For instance, about 63% of patients in the DME cohort had their fellow eye assessed to be the better eye at baseline, whereas approximately 88% of patients in the RVO cohort reported this for the fellow eye.

**Figure 4:**
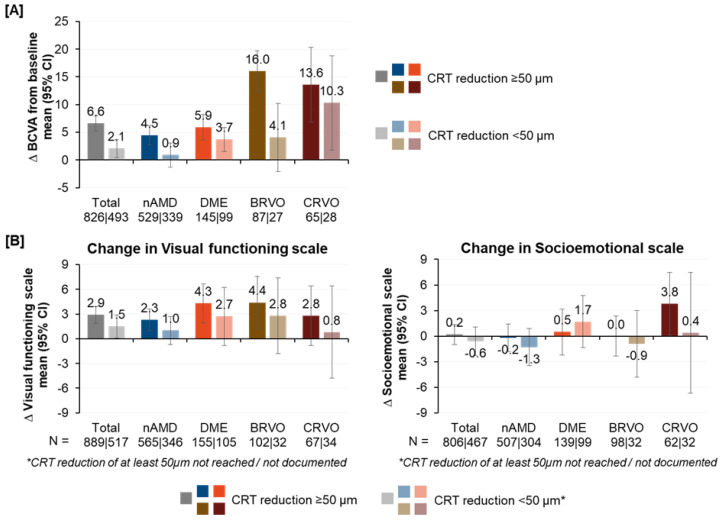
Association of CRT reduction on changes from baseline in BCVA (**A**) and NEI VFQ-25 VFS and SES (**B**) over twelve months. Higher scores indicated better visual acuity and QoL.

Overall, there was substantial decrease of central retinal thickness (CRT) after treatment initiation with anti-VEGF treatment as assessed by OCT (Supplementary Figure S3). The relationship between CRT reduction and changes in visual acuity in BCVA, as well as VFS and SES subscales, was evaluated, which revealed that CRT reductions of at least 50 µm were associated with greater improvements in visual acuity and NEI VFQ-25 subscales than reductions of less than 50 µm (Figure 4). Patients with nAMD and BRVO showed the largest association between CRT reduction and BCVA (Ratio_nAMD_: 5.0; Ratio_BRVO_: 3.9), while the patients with CRVO and DME showed a lower association (Ratio_CRVO_: 1.3; Ratio_DME_: 1.5). For patients with CRVO, CRT reduction was numerically associated with the largest improvements in VFS (RatioCRVO: 3.5) and SES (RatioCRVO: 9.5), with SES scores about one magnitude higher for CRVO patients with CRT reductions of at least 50 µm. Similarly, for nAMD and BRVO, higher CRT reductions were associated with higher SES scores (nAMD: −0.2 vs −1.3; BRVO: 0.0 vs. −0.9). However, in patients with DME, those with CRT reductions of at least 50 µm showed lower SES scores than patients with less than 50 µm reductions (SES scores, DME: 0.5 vs. 1.7), despite higher VFS scores (Ratio_DME_: 1.6).

The impact of routine vs decision-based OCT examinations on VRQoL was assessed by comparing results from the ALBATROS study to those from the non-interventional OCEAN study. The OCEAN study was conducted prior to the routine reimbursement of OCT examinations by the German statutory health insurance and therefore, such examinations were not routinely performed in clinical practice during that time.

For this purpose, the propensity score-matched datasets of ALBATROS and OCEAN were used. In both matched populations of ALBATROS and OCEAN, patients with nAMD exhibited substantially lower NEI VFQ-25 VFS and SES scores at baseline than those with DME or RVO.

**Figure 5:**
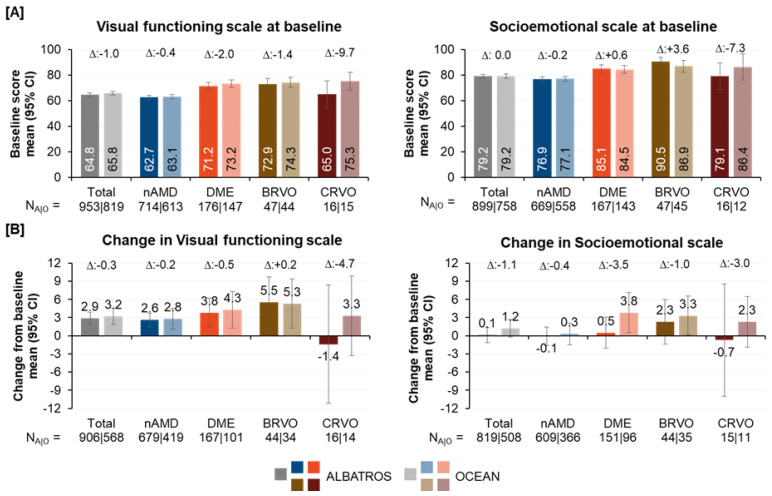
NEI VFQ-25 VFS and SES at baseline for ALBATROS and OCEAN matching datasets (**A**) and comparative analysis of changes in NEI VFQ-25 VFS and SES after twelve months (**B**). Higher scores indicate higher QoL.

The baseline scores for the overall, nAMD, and DME cohorts were similar between the ALBATROS and OCEAN matching populations varying by ≤2.0 points (Figure 5A). However, there were differences observed for the RVO cohorts. CRVO patients in the matched ALBATROS population showed 9.7 points lower VFS and 7.3 points lower SES compared to the corresponding OCEAN cohort. Conversely, BRVO patients in the matched ALBATROS cohort showed 3.6 points higher SES and only slightly lower VFS (−1.4 points) compared to the matched OCEAN cohort. It should be noted that both RVO populations had low samples sizes of less than 50 patients (BRVO) or less than 20 patients (CRVO) per cohort.

In general, there were differences in changes observed in NEI VFQ-25 VFS after twelve months among different indications, with higher gains in indications having higher baseline scores (Figure 5B). The magnitude of change in VFS after twelve months was similar between the matched ALBATROS and OCEAN cohort for the overall population (+2.9 and +3.2 points) as well as for nAMD, DME, and BRVO for indications varying by ≤0.5 points. Consistent with the results observed for the ALBATROS population, the matching ALBATROS population also showed the highest gains in VFS for patients with BRVO. Similarly, the matched OCEAN population also showed the highest gains in VFS for patients with BRVO. However, in contrast to the CRVO cohort of the ALBATROS population, which showed gains in VFS of 2.2 points after twelve months (see Figure 3A), CRVO patients in the matched ALBATROS population showed a loss of 1.4 points, and 4.7 points less than patients of the matching OCEAN cohort.

Changes in NEI VFQ-25 SES after twelve months were low and similar for the ALBATROS and OCEAN overall population and nAMD cohort. DME patients in ALBATROS exhibited a gain of 3.5 points less in SES score compared to the corresponding OCEAN cohort (0.5 points vs 3.8 points). For patients with BRVO, similar gains were observed for both the ALBATROS and OCEAN population (2.3 vs. 3.3). However, since BRVO patients of ALBATROS had higher baseline values compared to OCEAN (90.5 vs. 86.9), the difference might be underestimated. Similar to the VFS, CRVO patients in ALBATROS showed a loss of 0.7 points in SES, which was 3.0 points less than patients of the matching OCEAN cohort. This loss was not observed for the CRVO cohort of the ALBATROS population, which showed gain in SES of 2.7 points after twelve months (see Figure 3A).

Also, no substantial differences between the matched ALBATROS and OCEAN cohorts were observed for NEI VFQ-25 composite score after twelve months (Supplementary Figure S4).

## 4. Discussion

ALBATROS provides real-world data on the impact of mandatory OCT examinations on visual acuity and vision-related quality of life in the era before OCT was implemented in the SHI benefits catalogue. Published data of the non-interventional study OCEAN, which investigated treatment patterns with the anti-VEGF treatment ranibizumab and included optional OCT examinations, served as a reference, mirroring real-world evidence under less stringent OCT examination conditions. The most common treatment regimens in ALBATROS were ‘’treat and extend’’ or modifications of this regimen, such as ‘’monitor and extend,’’ and confirming observations made in other real-world studies [9,10]. The improvements in BCVA observed twelve months after treatment initiation with any approved anti-VEGF treatment were quite similar to reported values reported in current meta-analyses for DME, BRVO, and CRVO [11,12,13]. However, the improvements in BCVA for nAMD were lower in ALBATROS compared to those previously reported [14]. Improvements in visual acuity depend on several demographic and baseline factors, including age, baseline lesion size, and number of injections received, as well as the treatment regimen [1,15]. There was a trend towards higher BCVA improvements with higher OCT frequency for nAMD and the two RVO cohorts, but statistical significance was only obtained for the BRVO cohort. In settings where undertreatment is an issue [1,11], higher examination and OCT frequencies may be beneficial for patients’ visual outcomes. However, in addition to performing OCT examinations, a thorough examination and interpretation of the morphological results, as well as firm conclusions, are necessary. As reported by Liakopoulos et al. [16] in a previous study, a high number of performed OCT examinations indicating disease activity were misjudged by the treating physician and ultimately led to a decline in patients’ visual acuity. Therefore, the impact of OCT examination frequency on BCVA improvements may also be underestimated in ALBATROS. Vision functioning scale of NEI VFQ-25 improved in all indications by 3.1% to 5.0% compared to the respective baseline value. The magnitude of changes in socioemotional scale was 0.2% to 3.4%. Higher improvements for the VFS were observed for indications with higher baseline values but did not reflect gain in visual acuity for the particular indications. In respect to SES, observed changes were lower for study indication populations with a higher percentage of female patients. Observed values in ALBATROS for nAMD were slightly lower (0.6 points) compared to the range of 0.7 to 4.4 derived from a systematic literature review [17]. However, it currently seems unclear whether early improvements in VRQoL for nAMD are actually maintained, as different studies reported different outcomes [18]. For DME, BRVO and CRVO, the change in the observed composite score values in ALBATROS (DME: 2.3 points, BRVO: 0.8 points) were lower than those reported in a real-world setting [19] or RCTs [20,21] (DME: 2.9 points, BRVO: 7.2 points, CRVO: 6.2 to 7.5 points). 

Among the study indications, only DME patients showed an association between OCT examination frequency and improvement in NEI VFQ-25 VFS and SES. As for RVO patients, they experienced the highest gains in BCVA in the study eye, which makes the decrease in Rasch-transformed subscale scores with increasing number of OCT examinations for patients with RVO vs. other indications unexpected. This observation could be explained by the fact that the fellow eye had good and stable vision in most of these cases. Previous studies have shown that the visual acuity of the better eye has a high impact on VRQoL, although lower visual acuity in the fellow eye also shows an impact on VRQoL [22,23]. It is possible that worsening of visual acuity of a fellow eye, whose visual acuity was initially better than the study eye, leads to better adherence due to the fellow eye including more follow-up evaluations at the particular OCT visits. Thus, the higher number of OCT examinations for RVO patients might not necessarily be attributed to the study eye itself, but to the worsening of the fellow eye. If higher numbers of OCT examinations for RVO patients are actually correlated to the deterioration of the fellow eye, it might be causative for the lower VFS scores for patients with more OCT examinations. In general, reductions in CRT of at least 50 µm vs. CRT reductions of less than 50 µm were associated with improvement in visual acuity and in NEI VFQ-25 subscale scores. 

The comparison of VRQoL data of ALBATROS and OCEAN did not rule out a potential benefit of regular OCT examinations but failed to detect a difference that was also reflected (despite noise) in a higher VRQoL. A thorough examination and interpretation of the morphological results, as well as firm conclusion are important, even if the examinations were reliable/obligatory [16]. A limitation of the study is the lower proportion of included subjects with complete documentation. Nevertheless, it is one of the largest real-world samples investigating the impact of OCT monitoring and therapy on vision-related quality of life for various diseases with intravitreal injection therapy. Another limitation relates to the comparison of ALBATROS and OCEAN data, as the studies have different designs (data collection vs non-interventional study) and application of non-uniform anti-VEGF treatments (any anti-VEGF treatment vs. ranibizumab). The results of RVO cohorts should be interpreted with caution due to the low sample number, particularly of the CRVO cohort. All findings should be considered exploratory, and larger (randomized) studies are needed to confirm the results. Further, OCT angiography was not included in the study as it was not commercially available at the time of study development. 

In summary, the data suggest that the benefit of OCT examinations on visual acuity and VRQoL appears to be minor compared to the benefits of the anti-VEGF therapies themselves given to a patient. However, as higher CRT reductions were associated with better visual outcomes and VRQoL, timely and firm morphological assessments are essential to avoid undertreatment of patients and to exploit the full potential of currently available anti-VEGF therapies.

## Figures and Tables

**Table 1:** Demographics, baseline characteristics, and study metrics (CAS) of the ALBATROS study.

	Total	nAMD	DME	BRVO	CRVO
**Demographics and Baseline characteristics**					
Patients, n (%)	1478 (100)	964 (65.2)	272 (18.4)	140 (9.5)	102 (6.9)
Age, years	74.5 ± 10.9	78.3 ± 8.0	65.9 ± 12.3	68.7 ± 11.4	70.4 ± 11.8
Sex, female (%)	54.9	61.1	38.6	52.1	43.1
BCVA study [fellow] eye, letters	56.1 ± 19.6 [65.8 ± 25.0]	54.4 ± 19.6 [62.2 ± 27.2]	65 ± 14.2 [69.2 ± 20.4]	59.1 ± 19.1 [77.2 ± 13.9]	44.9 ± 23.1 [75.3 ± 16.4]
Better eye at baseline is fellow eye, n (%)	1028 (69.6)	643 (66.7)	172 (63.2)	123 (87.9)	90 (88.2)
CRT, µm	388.7 ± 150.3	373.5 ± 141.3	358.9 ± 114.5	458.4 ± 172.8	516.1 ± 189.8
NEI VFQ-25 *					
Composite score	73.5 ± 18.1	71.1 ± 18.3	77.4 ± 16.8	82.2 ± 12.4	73.6 ± 20.2
Visual functioning scale (VSF)	65.7 ± 20.9	62.8 ± 20.8	71.1 ± 20.1	74.4 ± 17.1	67.6 ± 22.5
Socioemotional scale (SES)	80.0 ± 22.0	77.3 ± 22.8	84.1 ± 19.4	90.7 ± 12.9	78.4 ± 24.2
**Study metrics**					
Number of OCT examinations	8.8 ± 2.6	8.7 ± 2.6	8.9 ± 2.9	8.9 ± 2.5	8.9 ± 2.6
Patients by number of OCT examinations, n (%)					
≤3 OCTs	22 (1.5)	14 (1.5)	4 (1.5)	1 (0.7)	3 (2.9)
>3 to ≤6 OCTs	299 (20.2)	194 (20.1)	64 (23.5)	26 (18.6)	15 (14.7)
>6 to ≤9 OCTs	515 (34.8)	343 (35.6)	78 (28.7)	53 (37.9)	41 (40.2)
>9 OCTs	642 (43.4)	413 (42.8)	126 (46.3)	60 (42.9)	43 (42.2)
Number of injections	6.1 ± 3.2	6.1 ± 3.1	6.1 ± 3.4	6.0 ± 3.2	6.0 ± 3.3
Number of injections by OCT examinations					
≤3 OCTs	2.2 ± 0.9	2.2 ± 1.0	1.5 ± 0.6	3.0 ± 0.0	3.0 ± 0.0
>3 to ≤6 OCTs	3.6 ± 1.6	3.7 ± 1.6	3.5 ± 1.8	3.5 ± 1.5	2.9 ± 1.7
>6 to ≤9 OCTs	5.4 ± 2.6	5.6 ± 2.6	4.9 ± 2.6	5.1 ± 2.5	5.4 ± 2.6
>9 OCTs	7.9 ± 3.1	7.7 ± 3.1	8.3 ± 3.2	8.0 ± 3.1	7.9 ± 3.2

Data are presented as mean ± SD, if not otherwise indicated; data presented for CAS. * Higher values indicated higher QoL.

## Data Availability

The data presented in this study are available on request from the corresponding author. The data are not publicly available due to intellectual property reasons.

## Supplementary Materials

The following supporting information can be downloaded at: https://www.mdpi.com/article/10.3390/jcm12123881/s1, Figure S1: STROBE flow chart of patient disposition; Figure S2: NEI-VFQ25 subscale and composite scores of the total population and nAMD, DME, BRVO and CRVO cohorts at baseline and after twelve months. Higher scores indicate higher QoL; Figure S3: Central retinal thickness (CRT) at baseline and subsequent time points for total population, nAMD-, DME-, BRVO- and CRVO cohorts. Vertical bars present number of available patients per month; colored shadings present 95% confidence intervals; numbers in the figure indicate CRT after twelve months. Note different scaling of CRT and number of available patients for particular indications; Table S1: Results from logistic regression for propensity score matching of ALBATROS and OCEAN data; Figure S4: NEI-VFQ25 composite scores of ALBATROS and OCEAN total population, nAMD-, DME-, BRVO- and CRVO cohorts at baseline [A] and after twelve months [B]. Higher scores indicate higher QoL.
