# Supplementary material for: Impact of Routinely Performed Optical Coherence Tomography Examinations on Quality of Life in Patients with Retinal Diseases—Results from the ALBATROS Data Collection
Source: J Clin Med. 2023 Jun 7;12(12):3881. doi: 10.3390/jcm12123881 (PMC10299675; doi:10.3390/jcm12123881)

Figure S1. STROBE flow chart of patient disposition

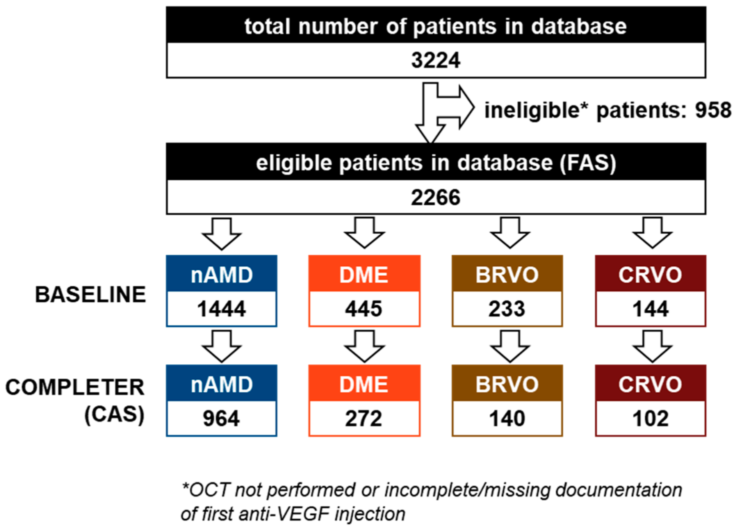

Supplement: Supplementary file 1 [file jcm-12-03881-s001.zip › Figure S1.pdf]
