# Supplementary material for: Impact of Routinely Performed Optical Coherence Tomography Examinations on Quality of Life in Patients with Retinal Diseases—Results from the ALBATROS Data Collection
Source: J Clin Med. 2023 Jun 7;12(12):3881. doi: 10.3390/jcm12123881 (PMC10299675; doi:10.3390/jcm12123881)

Figure S3. Central retinal thickness (CRT) at baseline and subsequent time points for total population, nAMD-, DME-, BRVO- and CRVO cohorts. Vertical bars present number of available patients per month; colored shadings present 95% confidence intervals; numbers in the figure indicate CRT after twelve months. Note different scaling of CRT and number of available patients for particular indications.

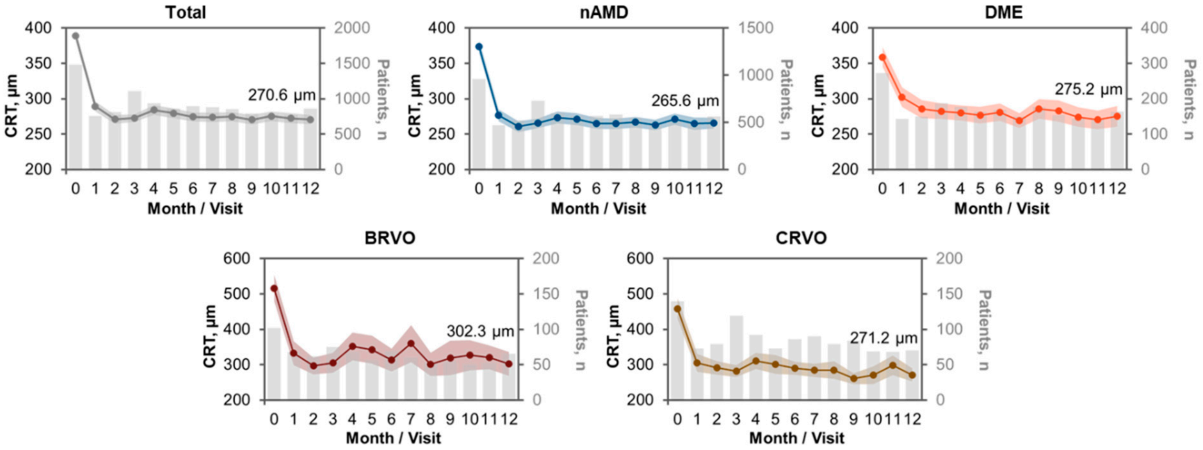

Supplement: Supplementary file 1 [file jcm-12-03881-s001.zip › Figure S3.pdf]
