# Supplementary material for: Impact of Routinely Performed Optical Coherence Tomography Examinations on Quality of Life in Patients with Retinal Diseases—Results from the ALBATROS Data Collection
Source: J Clin Med. 2023 Jun 7;12(12):3881. doi: 10.3390/jcm12123881 (PMC10299675; doi:10.3390/jcm12123881)

Figure S4. NEI-VFQ25 composite scores of ALBATROS and OCEAN total population, nAMD-, DME-, BRVO- and CRVO cohorts at baseline [A] and after twelve months [B]. AMAS, ALBATROS matching set; OMAS, OCEAN matching set. Higher scores indicate higher QoL.

[A]

AMAS (ALBATROS)	Total	nAMD	DME	BRVO	CRVO
Patients, n	955	716	176	47	16
NEI-VFQ25 Composit score at baseline	72.7±18.45	70.9±18.73	77.6±17.14	81.7±11.29	72.9±20.59
**OMAS (OCEAN)**	**Total**	**nAMD**	**DME**	**BRVO**	**CRVO**
Patients, n	837	625	150	46	16
NEI-VFQ25 Composit score at baseline	73.3±18.95	71.3±19.79	78.8±16.01	80.2±10.22	80.6±13.51

Data are presented as mean±SD, if not otherwise indicated

[B]

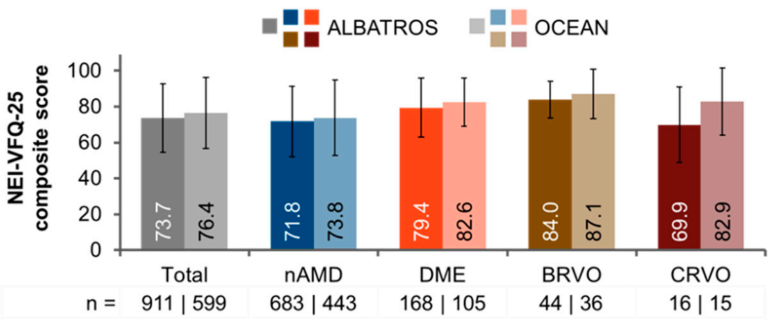

Supplement: Supplementary file 1 [file jcm-12-03881-s001.zip › Figure S4.pdf]
