# Supplementary material for: Impact of Routinely Performed Optical Coherence Tomography Examinations on Quality of Life in Patients with Retinal Diseases—Results from the ALBATROS Data Collection
Source: J Clin Med. 2023 Jun 7;12(12):3881. doi: 10.3390/jcm12123881 (PMC10299675; doi:10.3390/jcm12123881)
Supplement: Supplementary file 1 [file jcm-12-03881-s001.zip › Table S1.pdf]

Table S1. Results from logistic regression for propensity score matching of ALBATROS and OCEAN data

Model variable included	Estimate	Standard error	Wald χ^2	Prob. > χ^2
Age (categories)	0.0015	0.0202	0.0057	0.9398
Sex	-0.0008	0.0689	0.0001	0.9904
Primary indication	0.2511	0.0411	37.3271	<0.0001
Hypertonia	1.3880	0.0680	416.4243	<0.0001
BCVA (logMAR) of study eye at baseline visit	-0.2118	0.0910	5.4211	0.0199
